# Cardiovascular changes in patients with acromegaly assessed by CMR

**DOI:** 10.1186/1532-429X-14-S1-O85

**Published:** 2012-02-01

**Authors:** Filip Zemrak, Julia Thomas, Abhishek Dattani, Thomas R Burchell, Steffen E Petersen, Ashley Grossman, Marta Korbonits, Ceri Davies

**Affiliations:** 1 Cardiovascular Biomedical Research Unit, Barts and the London School of Medicine and Dentistry, Queen Mary University of London, London, UK; 2 The London Chest Hospital, London, UK; 3 Centre for Endocrinology, William Harvey Research Institute, Barts and the London School of Medicine and Dentistry, Queen Mary University of London, London, UK; 4 Oxford Centre for Diabetes, Endocrinology and Metabolism, University of Oxford, London, UK

## Summary

The study describes cardiovascular changes in patients with acromegaly before and one year after treatment.

## Background

Acromegaly causes a distinct cardiomyopathy, which remains poorly understood, because cardiac changes typically appear before the development of hypertension or diabetes.

The aim of the study was to describe cardiovascular changes in patients with acromegaly before and one year after treatment.

## Methods

Thirteen patients with acromegaly and age- and sex-matched controls (n=13) underwent CMR. Patients underwent scans before disease treatment and at twelve months after treatment. Cardiac parameters were calculated and indexed to body surface area (BSA). The comparison between groups was done using Mann-U-Whitney test and within the group using Wilcoxon test.

## Results

In patients with acromegaly left ventricular (LV) mass index (LVMi) was increased (65.7 vs. 45.8 g/m2, p=0.0021) and was observed in both females (58.8 v. 40.9 g/m2, p=0.0028) and males (71.1 vs. 56.7 g/m2, p=0.0286) compared to matched controls. The LVMi did not correlate with the serum insulin growth factor (IGF) activity (r=0.099, p=0.745) or age (r=-0.08, p=0.175).

Patients with acromegaly had significantly higher cardiac index (CI; 3.7 vs. 3.0 l/min/m2, p=0.021) However, there were no differences between end diastolic volume index (EDVi; 86.9 vs. 75.4 ml/m2, p=0.0649), end systolic volume index (ESVi; 35.1 vs. 29.3 ml/m2, p=0.1662) and ejection fraction (EF; 60 vs. 59 %, p=0.327) in acromegaly group and controls.

There were no differences between right ventricular (RV) RVEDVi (81.3 vs. 72.5 ml/m2, p=0.2382), RVESVi (32.7 vs. 29.1, p=0.6816) and RVEF (61 vs. 59 %, p=0.4407) in the acromegaly group and controls.

At one year, patients with acromegaly demonstrated a significant fall in IGF with treatment (with somatostatin analogues or transphenoidal surgery) from baseline median IGF-I SDS +10.58 (range 1.19 to 6.52) to +0.40 (range -1.93 to 3.02) at one year (p=0.0042). CMR parameters of the LV did not change after 1 year of therapy: LVMi 65.7 vs. 61.0 g/m2, p=0.0547; EDVi 89.5 vs. 85.8 ml/m2, p=0.1641; ESVi 33.7 vs. 30.1 ml/m2, p=0.6523; EF 60 vs. 66 %, p=0.7792; CI 3.7 vs. 3.4 l/min/m2, p=0.4961.

## Conclusions

Left ventricular mass and cardiac index are increased in patients with acromegaly. The degree of hypertrophy is not correlated with age or insulin growth factor activity. The lack of improvement of the left ventricular mass after 12 months of therapy may be a reflection of incomplete acromegaly treatment and will require further studies.

## Funding

The study has been supported by departmental grants from Pfizer and Novartis.

